# Surveillance of avian influenza viruses in live bird markets of Shandong province from 2013 to 2019

**DOI:** 10.3389/fmicb.2022.1030545

**Published:** 2022-11-03

**Authors:** Ti Liu, Yousong Peng, Julong Wu, Shangwen Lu, Yujie He, Xiyan Li, Lin Sun, Shaoxia Song, Shengyang Zhang, Zhong Li, Xianjun Wang, Shu Zhang, Mi Liu, Zengqiang Kou

**Affiliations:** ^1^Shandong Provincial Key Laboratory of Infectious Disease Control and Prevention, Shandong Center for Disease Control and Prevention, Jinan, China; ^2^Bioinformatics Center, College of Biology, Hunan Provincial Key Laboratory of Medical Virology, Hunan University, Changsha, China; ^3^Chinese National Influenza Center, National Institute for Viral Disease Control and Prevention, Chinese Center for Disease Control and Prevention, Beijing, China; ^4^Jiangsu Institute of Clinical Immunology, The First Affiliated Hospital of Soochow University, Suzhou, China

**Keywords:** avian influenza viruses, surveillance, H9N2 AIV, epidemic, genotypes

## Abstract

Avian influenza viruses (AIVs) seriously affect the poultry industry and pose a great threat to humans. Timely surveillance of AIVs is the basis for preparedness of the virus. This study reported the long-term surveillance of AIVs in the live bird market (LBM) of 16 cities in Shandong province from 2013 to 2019. A total of 29,895 samples were obtained and the overall positive rate of AIVs was 9.7%. The H9 was found to be the most predominant subtype in most of the time and contributed most to the monthly positve rate of AIVs as supported by the univariate and multivariate analysis, while H5 and H7 only circulated in some short periods. Then, the whole-genome sequences of 62 representative H9N2 viruses including one human isolate from a 7-year-old boy in were determined and they were genetically similar to each other with the median pairwise sequence identities ranging from 0.96 to 0.98 for all segments. The newly sequenced viruses were most similar to viruses isolated in chickens in mainland China, especially the provinces in Eastern China. Phylogenetic analysis showed that these newly sequenced H9N2 viruses belonged to the same clade for all segments except PB1. Nearly all of these viruses belonged to the G57 genotype which has dominated in China since 2010. Finally, several molecular markers associated with human adaptation, mammalian virulence, and drug resistance were identified in the newly sequenced H9N2 viruses. Overall, the study deepens our understanding of the epidemic and evolution of AIVs and provides a basis for effective control of AIVs in China.

## Introduction

The influenza A virus belongs to the *Orthomyxoviridae* family and contains a negative-sense RNA genome with eight segments. The influenza A virus is classified into different subtypes based on the surface proteins of haemagglutinin (HA) and neuraminidase (NA), such as H7N9, H5N1, or H9N2. The natural hosts of the influenza A virus are wild birds including both the wild waterfowl and sea birds (Webster et al., 1992). The avian influenza viruses (AIVs) are influenza A viruses that mainly infect the avians including both wild birds and poultry. The AIVs have caused numerous epidemics in poultry globally and have seriously affected the poultry industry (Su et al., 2015). Besides, they can occasionally cause human infections (Mostafa et al., 2018). Lots of subtypes of AIVs have been reported to infect humans in recent years, such as H5N1, H5N6, H7N9, H9N2, H10N8, H5N8, H3N8, and so on (Mostafa et al., 2018; Li et al., 2019b). How to effectively control AIVs is a great challenge for humans.

China is among the countries with the most diverse AIVs as the country has a large number of wild bird species and maintains the largest number of poultry in the world (Liu S. et al., 2020). Multiple subtypes of AIVs have circulated extensively in China in the last 20 years. Among them, the subtypes of H5, H7, and H9 are most predominant in China (Su et al., 2015; Liu S. et al., 2020). The subtype H5 has circulated in China for more than 20 years since several large-scale outbreaks occurred in 2001 which were caused by the highly pathogenic avian influenza (HPAI) H5N1 virus (Peng et al., 2017). The HPAI H5N1 virus has ever been considered to be most likely to cause global pandemics before 2009 when the H1N1 pandemic happened. It was almost the exclusive subtype among H5 subtypes which circulated in China before 2012 (Liu S. et al., 2020). Then, the subtypes of H5N2, H5N6, and H5N8 emerged and replaced the HPAI H5N1 virus in China. The subtype H7 has been widely circulating in China since 2013 when the H7N9 virus caused human infections (Jiang et al., 2019). Until now, the H7N9 virus has caused more than 1,500 confirmed human infections and more than 500 human deaths in China (Quan et al., 2018). The virus has caused multiple outbreaks in chickens since it evolved into the HPAI virus in 2016. Fortunately, the virus is now rarely detected in China because of the simultaneous immunization of the H5 + H7 vaccine among poultry since 2017 (Li and Chen, 2021).

Compared to subtypes of H5 and H7, the H9 subtype was the first AIV subtype that caused widespread infections in poultry in China (Peacock et al., 2019; Liu S. et al., 2020). The H9N2 virus has been circulating in China since the 1990s. Several large-scale surveillance studies have shown that the H9N2 virus was the most prevalent subtype in poultry in China (Bi et al., 2020). The virus also caused sporadic human infections, most of which happened in poultry workers. The H9N2 virus in China could be classified into three large clades, i.e., BJ/94, G1, and F/98 based on epidemiological and phylogenetic analysis. Li et al. further classified the H9N2 virus in China into at least 117 genotypes by evolutionary analysis (Li et al., 2017). Among them, the G57 genotype has become dominant in China since 2010 (Peacock et al., 2019). Due to the large diversity and high prevalence in birds, the H9N2 virus plays an important role in the evolution of AIVs by providing internal genes to other AIVs, which lead to novel AIVs (Peacock et al., 2019). For example, the H7N9 virus which has caused human infections since 2013 was reported to obtain all its internal genes from H9N2 viruses by re-assortment (Wu et al., 2013).

Shandong is a big agricultural province of China and has a developed poultry industry which poses a high risk of AIV outbreaks. Timely surveillance of AIV is the basis for better preparedness of the virus. However, there was little data about the epidemiology and evolution of AIVs in the province in recent years. This study reported the long-term surveillance of AIVs in the live bird market (LBM) of 16 cities in Shandong province from 2013 to 2019. The H9 was found to be the most predominant subtype during the period. Thus, the whole-genome sequences of 62 representative H9N2 viruses including 61 environmental isolates and one human isolates were determined by the next-generation-sequencing method. The evolution of these viruses and the molecular markers they contained were further analyzed. The study deepens our understanding of the epidemic and evolution of AIVs and provides a basis for effective control of AIVs in China.

## Materials and methods

### Virus sampling and isolation

A total of 29,895 samples were obtained from the environments including the surface wipe of poultry cages, chopping boards, poultry drinking water and feces, of LBMs in 16 cities of Shandong province, China, from 2013 to 2019. The samples were placed in the 3 ml of Viral Transport Medium (VTM), and then were centrifuged at 3000 g for 10 min. The supernatants were used to extract RNA with the Qiagen RNeasy Mini Kit (Lot. 74,104) according to the manufacturer’s instructions. The real-time RT-PCR was used to detect AIVs. If the sample was positive for AIVs, the sample was further subtyped for H5, H7 and H9. The positive samples of H9 subtype were inoculated into the allantoic cavity of 9-day-old specific pathogen-free embryonated chicken eggs and incubated for 72 h at 37°C and chilled at 4°C overnight. The allantoic fluids were harvested and the influenza A(H9N2) virus strains were identified with a combination of hemagglutination assay with horse erythrocytes and real-time RT-PCR of H9N2 detection.

### Human H9N2 case finding and isolation

On April 28th, 2020, a 7-year-old boy living in Weihai, a city in Shandong province, was taken to Weihai Municipal Hospital for influenza-like illness. A nasopharyngeal sample obtained was tested positive for influenza A and H9N2 at the Weihai Center for Disease Control and Prevention. The sample was then sent to the Shandong Provincial Center for Disease Control and Prevention,and the virus (A/human/shandong/01/2020) was successfully isolated in embryonated chicken eggs and confirmed as H9N2.

### Genome sequencing

A total of 62 H9N2 strains including 61 environmental strains isolated in the surveillance efforts and one human isolate mentioned above were sequenced by the next-generation-sequencing method (Supplementary Table S1). The total viral RNA of these strains was extracted with the Qiagen RNeasy Mini Kit (Lot. 74,104). The RNA was subjected to reverse transcription and amplification using the SuperScript™ III One-Step RT-PCR System with Platinum™ Taq High Fidelity DNA Polymerase (cat#: 12574035, Invitrogen). The DNA library was prepared using Nextera XT DNA Preparation Kits (cat#FC-131-1,096, Illumina). Whole-genome sequencing was then performed on MiSeq high-throughput sequencing platform (Illumina, Inc., San Diego, CA, United States), and the data were analyzed using CLC Genomics Workbench software.

### Phylogenetic analysis

Except for newly sequenced H9N2 viruses, we also collected other H9N2 viruses from the public database for phylogenetic analysis. The nucleotide sequences of H9N2 viruses were downloaded from the database of Influenza Virus Resource on October 25th, 2021 (Bao et al., 2008), and were clustered using CD-HIT to create the reference sequence database (Li and Godzik, 2006). The representative viruses together with the newly sequenced viruses were used to build phylogenetic trees. Phylogenetic trees for all eight segments of H9N2 viruses were generated by the maximum likelihood method using MEGA X (Kumar et al., 2018). The neighbor virus of the newly sequenced virus on each segment was identified by querying against the reference sequence database using BLASTN (version 2.13.0) (Altschul et al., 1997). The virus strain with the highest bit score was selected as the neighbor virus of the query sequence.

### Genotype determination

The genotypes of newly sequenced H9N2 viruses were determined based on the phylogenetic analysis according to previous studies (Pu et al., 2015; Li et al., 2017; Jin et al., 2020).

### Identification of molecular markers in H9N2 viruses

The human-adaptation, mammalian virulence and drug-resistance associated molecular markers in newly sequenced H9N2 viruses were identified with FluPhenotype on July 12th, 2022 (Lu et al., 2020).

### Statistical analysis

The univariate and multivariate analysis of the monthly positive rate of influenza viruses by subtype was conducted using the “lm” function in R (version 3.6.1).

## Results

### Surveillance of AIVs in LBMs of Shandong province

**Figure 1:**
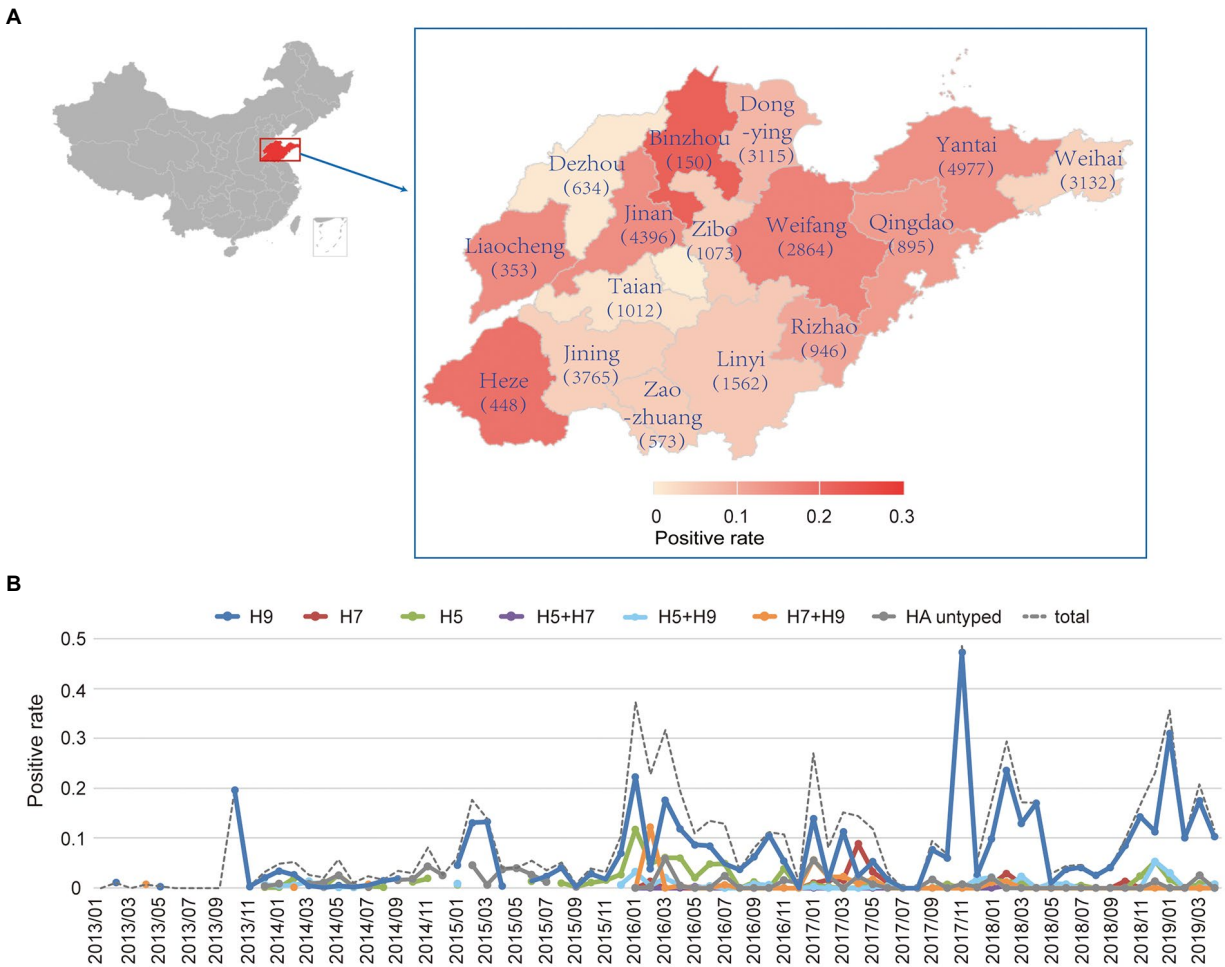
Surveillance of AIVs in LBMs of Shandong province from 2013 to 2019. **(A)** The number of samples and positive rates of AIVs by city in Shandong province. The cities were colored by the positive rate. The numbers in parentheses referred to the number of samples surveyed in the city. **(B)** The monthly positive rate of different HA subtypes or subtype combinations of influenza A viruses.

A total of 29,895 samples from the environments such as the surface wipe of poultry cages, poultry drinking water and feces in LBMs of 16 cities in Shandong province, China, were obtained from January 2013 to April 2019 (Figure 1A). Among them, 2,903 samples were positive for AIVs and the overall positive rate was 9.7%. When analyzed by city, the number of samples surveyed ranged from 150 to 4,977 in 16 cities. The positive rate ranged from 1 to 22% in these cities, with Binzhou, Heze and Weifang having the highest positive rates (Figure 1A).

The overall monthly positive rate of AIVs ranged from 0 to 0.485 with a median of 0.052 from January 2013 to April 2019 (Figure 1B and Supplementary Table S2). When analyzed by HA subtype, the monthly positive rates of H5, H7 and H9 were calculated from 2013 to 2019. Attention to note, the positive rate during the period of 2013–2015 may be underestimated as a large portion of samples was un-subtyped during the period. Both the univariate and multivariate analysis showed that the H9 subtype contributed most to the monthly positive rate (Table 1), while other HA subtypes or subtype combinations contributed little. The year and month had minor positive and negative effects, respectively, on the monthly positive rate in the univariate analysis, although both had no statistical significance. Interestingly, they became statistically significant in the multivariate analysis, suggesting complex interactions between subtype, year and month.

**Table 1:** Univariate and multivariate analysis of the monthly positive rate by subtype.

Variable	Univariate analysis			Multivariate analysis		
	Coefficient	Standard Error	*p*-value	Coefficient	Standard Error	*p*-value
*Subtype*						
H9	0.060	0.007	2.39E-16	0.061	0.007	<2.2E-16
H7	−0.005	0.008	0.509			
H5	0.003	0.007	0.651			
H5&H7	−0.011	0.008	0.169			
H5&H9	−0.005	0.007	0.504			
H7&H9	−0.004	0.008	0.576			
Year	0.0006	0.002	0.714	0.004	0.001	0.0098
Month	−0.001	0.0007	0.070	−0.001	0.0006	0.042

As shown in Figure 1B, H9 was the main subtype in most of the time (Figure 1B), which was consistent with the univariate and multivariate analysis. It persisted circulating throughout the year from 2013 to 2019. In general, the H9 peaked in the winters and maintained a low level of circulation in the summers. H5 mainly circulated during the season of 2015–2016. It caused sporadic infections in some months. H7 circulated least compared to H5 and H9. It only dominated in the April of 2017 when 20 cases of human infections of the H7N9 virus were reported in Shandong province in the same year (Zhang et al., 2020), and maintained a very low level of activity in most of the time.

Besides the positive rates of individual HA subtypes, we also surveyed the co-occurrence of H5, H7, and H9 during the period. As expected, most co-occurrences happened between H9 and H5 or H7, and the co-occurrences were observed when both HA subtypes had high positive rates such as the co-occurrence of H9 and H5 observed in early 2016. Only a few co-occurrences of H5 and H7 were observed from 2013 to 2019 (Figure 1B).

### Sequencing and phylogenetic analysis of representative H9N2 viruses

**Figure 2:**
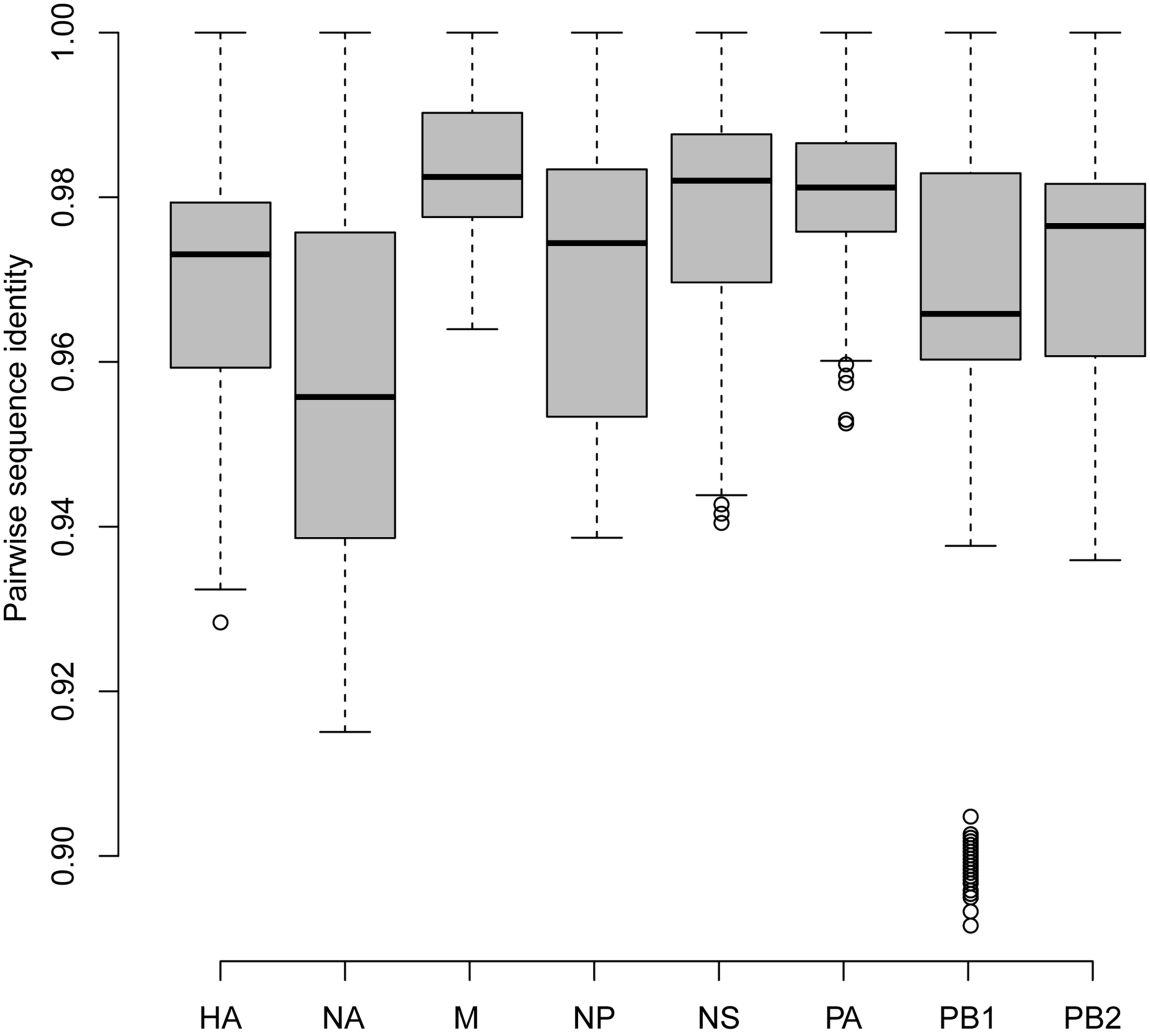
Pairwise sequence identities of each segment between newly sequenced H9N2 viruses.

Since the H9 was the most dominant subtype in our surveillance, the whole genome sequences of 62 representative H9N2 strains including 61 environment strains and one human isolate were obtained using the next-generation-sequencing method for better understanding the genetic evolution of H9N2 viruses in Shandong province (Supplementary Table S1). As shown in Figure 2, for all segments, the median pairwise sequence identities ranged from 0.96 to 0.98. The gene M had the highest pairwise sequence identities, while the NA gene had the lowest pairwise sequence identities. The HA gene had pairwise sequence identities ranging from 0.93 to 1, with a median of 0.97.

**Figure 3:**
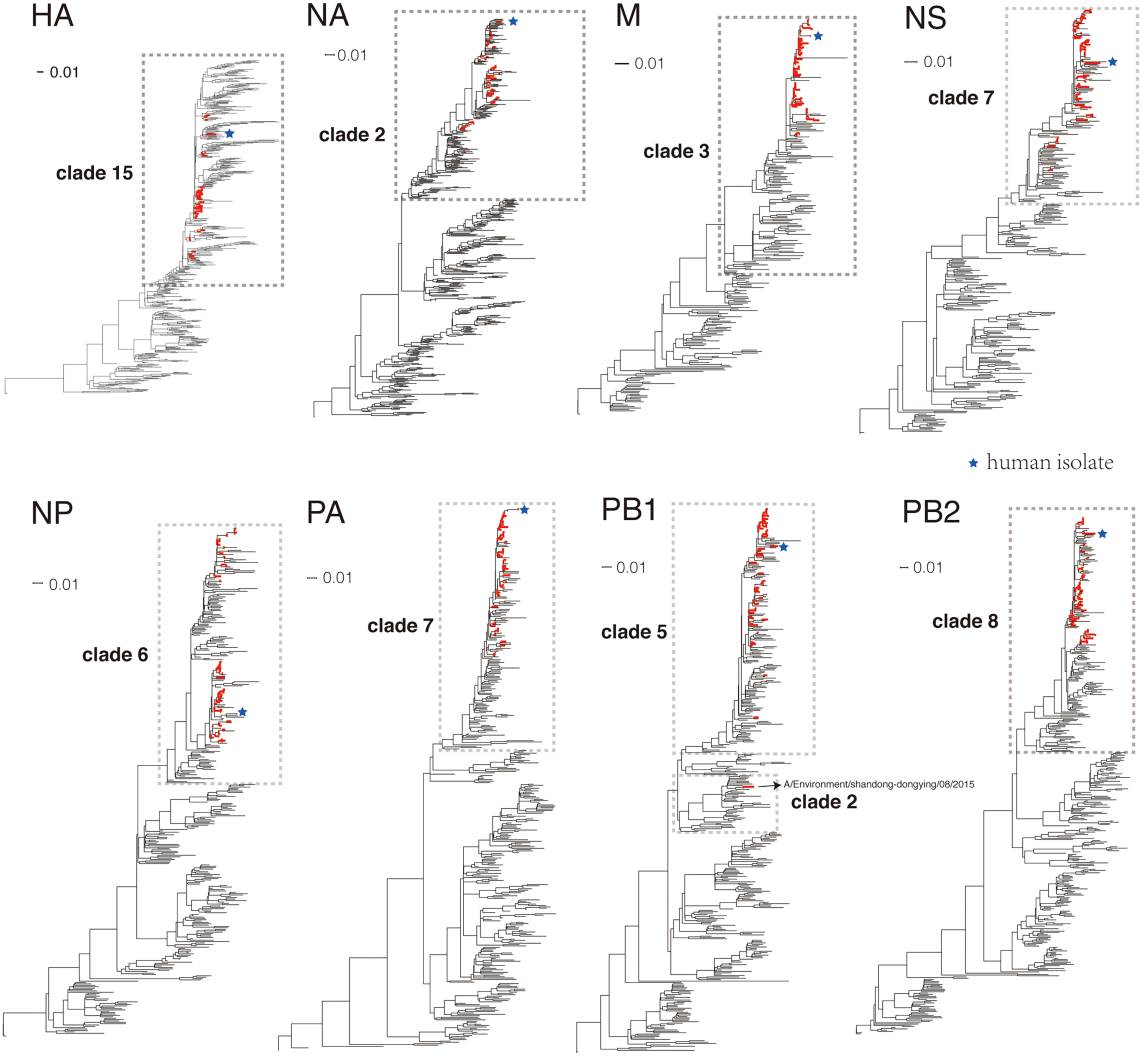
The phylogeny of H9N2 viruses on each segment. The newly sequenced H9N2 viruses were colored in red in the trees. The human isolate was marked with blue stars. The details of these trees were shown in Supplementary Figure S1.

These viruses were further genetically characterized by the phylogenetic analysis. For each segment of H9N2 viruses, several reference viruses of influenza H9N2 viruses were selected from the Influenza Virus Resource database and were used in phylogenetic analysis with the newly sequenced H9N2 viruses (see Materials and Methods). As shown in Figure 3, for all segments except PB1, all newly sequenced H9N2 strains (colored in red) belonged to the same clade (marked with a dashed box). For PB1, all viruses belong to clade 5 except the viral isolate A/Environment/shandong-dongying/08/2015 which belonged to clade 2 and was clustered with sequences isolated from wild birds (Supplementary Figure S1).

**Figure 4:**
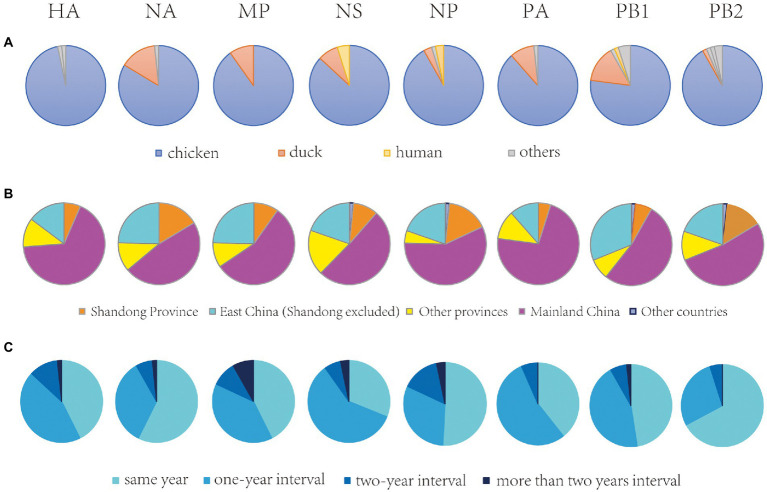
The host, isolation location and year composition of the neighbors of newly sequenced H9N2 viruses isolated in the environment.

We then investigated the possible source of the newly sequenced H9N2 viruses since all of them except the human isolate were isolated from the environment. For each segment of each virus, the neighbor of the virus (defined as the most similar virus) was obtained (see Materials and Methods), and the composition of the host, isolation location and year of the neighbors were analyzed (Figure 4). In terms of host, for all segments, most neighbors were isolated from the poultry including chicken and ducks, especially the chicken. In terms of isolation location, nearly all neighbors were isolated from mainland China, especially the Shandong province and its neighboring provinces in Eastern China such as Anhui, Jiangsu, and Zhejiang provinces. In terms of isolation time, most neighbors were isolated in the same year with, or 1 year before or after the year when the virus was isolated.

The neighbors of the human isolate A/human/shandong/01/2020 were analyzed individually. In terms of host, the neighbors of the human isolate were isolated from chicken for all segments; in terms of isolation location, for five segments (HA, NA, MP, PA and PB2), the neighbors were isolated in Anhui province, while for NP, NS and PB1, the neighbors were isolated in Fujian, Jiangxi and Shanxi province, respectively; in terms of isolation time, the neighbors of the human isolate were isolated in 2018 for all segments except NA of which the neighbor was isolated in 2019.

### Genotyping of newly sequenced H9N2 viruses

The genotypes of newly sequenced H9N2 viruses were determined based on the phylogenetic analysis according to Li’s study (Supplementary Table S3) (Materials and Methods). All viruses except A/Environment/shandong-dongying/08/2015 belonged to the G57 genotype which was first found in Eastern China in 2007 and has been the predominant genotype in China since 2010 (Li et al., 2017). The A/Environment/shandong-dongying/08/2015 belonged to G69 genotype which has a different PB1 clade compared to G57 and was rarely reported in China.

### Molecular markers of H9N2 viruses

Finally, we investigated the molecular markers associated with human adaptation, mammalian virulence, and drug resistance in the newly sequenced H9N2 viruses using FluPhenotype (Lu et al., 2020) (Materials and Methods). In terms of human adaptation, 11 molecular markers that were located in 7 proteins were observed, and 7 of them happened in more than 50 viruses (Table 2). For example, Threonine on 180 of the HA protein that was reported to be associated with an increase in binding to the human-like receptor was observed in 51 of 62 newly sequenced H9N2 viruses. The human isolate A/human/shandong/01/2020 had 6 human-adaptation associated molecular markers including HA-180 T, HA2-46E, PA-356R, PB1-368 V, PB1-F2-47S and PB2-66 M.

**Table 2:** The human-adaptation-related molecular markers identified in the newly sequenced H9N2 virus strains.

Protein	Mutation	Ratio	Mutation effect
HA	**180 T**	51/62	Increase in binding to the human-like receptor
	**HA2-46E**	62/62	Enhanced binding to both avian-and human-type receptor
M2	19Y	2/62	The human-adaptation associated residues
NP	109 V	1/62	The human-adaptation associated residues
PA	57Q	1/62	The human-adaptation associated residue
	100A	2/62	The human-adaptation associated residue
	**356R**	62/62	The human-adaptation associated residue
PB1	**368 V**	55/62	The human-adaptation associated residue
PB1-F2	**47S**	62/62	The human-adaptation associated residue
PB2	**66 M**	61/62	The human-adaptation associated residue
	**89 V**	62/62	The human-adaptation associated residue

Those which were found in more than 50 viruses were highlighted in bold.

In terms of mammalian virulence, 30 molecular markers that were located in 6 proteins were observed and 18 of them happened in more than 50 viruses (Table 3). For example, three markers in the MP1 protein including 30D, 43 M and 215A which were reported to increase virulence in mammals were observed in all 62 newly sequenced H9N2 viruses. The human isolate A/human/shandong/01/2020 had 6 molecular markers associated with mammalian virulence including MP1-30D, MP1-43 M, MP1-215A, NS1-42S, PA-224S and PB2-431 M.

**Table 3:** The mammalian-virulence-related molecular markers identified in the newly sequenced H9N2 strains.

**Gene**	**Mutation**	**Ratio**	**Mutation effect**
MP1	**30D**	62/62	Increased virulence in mice
	**43 M**	62/62	Contribute to the pathogenicity of HPAI H5N1 viruses in both avian and mammalian hosts
	**215A**	62/62	Increased virulence in mice
NS1	**42S**	61/62	Increased virulence in mice
	123 V	5/62	Selective advantage in replication and/or transmission of pH1N1 in humans
	127 N	40/62	Associated with high-virulence in mammals
PA	**37S**	62/62	Decreased viral transcription and replication by diminishing virus RNA synsthesis activity
	**37&61ST**	62/62	Decreased viral transcription and replication by diminishing virus RNA synthesis activity
	**63I**	62/62	Decreased viral transcription and replication by diminishing virus RNA synthesis activity
	70&224VS	17/62	Reducing the virus LD50 in mice by almost 1,000-fold
	**190S**	62/62	Reduced the virulence of H7N3 virus
	**224S**	62/62	Enhanced polymerase activity and virulence of pH1N1 in mice
	**400P**	62/62	Reduced the virulence of H7N3 virus
	409S	2/62	Increased virus replication ability in mammalian systems
	**550 l**	62/62	Increased polymerase activity and high virulence.
PB1	**13P**	62/62	Enhanced polymerase activities of 270%
	**317I**	56/62	I at 317 in PB1 correlated with high pathogenicity.
PB1-F2	51&56&87TVE	1/62	Increased viral polymerase activity and expression levels of viral RNA
PB2	195 N	5/62	The PB2-D195 N substitution increased polymerase activity by about 3.5-fold
	283&526MR	3/62	Enhanced virulence of H5N8 influenza viruses in mice
	**292 V**	54/62	Higher viral polymerase activity and stronger attenuation of host IFN-Î2 response
	**309D**	62/62	Increased polymerase activity
	**431 M**	62/62	Impacts the viral replication and virulence in mice by altering the viral polymerase activity
	**504 V**	62/62	Mutational analyses demonstrated that an isoleucine-to-valine change at position 504 in PB2 was the most critical and strongly enhanced the activity of the reconstituted polymerase complex.
	526R	3/62	Increased polymerase activity/enhanced pathogenicity in mice
	535 l	12/62	Increased polymerase activity
	598I	10/62	Increased virus replication and virulence in mice
	**661A**	60/62	Increased polymerase activity at low temperature
	702R	11/62	Increased polymerase activity / enhanced pathogenicity in mice
	89&309&339&477&495&627&676 VDKGVET	1/62	Increased virulence in mice.

Those which were found in more than 50 viruses were highlighted in bold.

In terms of drug resistance, 4 molecular markers which were located in NA and MP2 proteins were observed (Supplementary Table S4). The molecular marker NA-151D which was reported to be associated with resistance to oseltamivir and zanamivir, and molecular markers of MP2-21G and MP2-31 N which were reported to be associated with resistance to amantadine were observed in more than 60 newly sequenced H9N2 strains.

## Discussion

The LBM has been reported to play an important role in the spreading of AIVs because numerous poultry are transported in and out of LBMs (Cardona et al., 2009; Yu et al., 2014; Li et al., 2018). Studies have shown that closing the LBM had a large impact on the human infection of AIVs such as H7N9 viruses (Yu et al., 2014; Li et al., 2018). Besides, the LBM was also considered to be a vessel for mixing AIVs (Cardona et al., 2009). Lots of novel viruses can be generated in the LBM by re-assortment such as the H7N9 virus which has caused human infections since 2013 (Wu et al., 2013). In this study, we surveyed the AIV in LBMs of Shandong province from 2013 to 2019 and found persistent circulations of AIVs in the province. The H9 was found to be the main subtype in most of the time, while the H5 and H7 only dominated in some short periods. These were consistent with previous studies which showed the predominant role of H9 in China (Peacock et al., 2019; Liu S. et al., 2020). Our analysis also found significant co-occurrence of H5, H7 and H9, suggesting the high risk of re-assortment of AIVs in the LBM. Therefore, continual surveillance of AIVs in the LBM is required for the timely identification of novel flu viruses.

Vaccination is the best way for controlling the avian influenza virus. Large scale vaccination of poultry has been conducted in China for prevention and control of the avian influenza viruses including H5, H9 and H7 (Liu S. et al., 2020). On one hand, vaccination can greatly stop the spreading of avian influenza virus. For example, massive vaccination of chickens with an H5/H7 bivalent avian influenza vaccine since September 2017 has successfully controlled H7N9 avian influenza infections in poultry (Li and Chen, 2021). Our surveillance also showed that few H5 or H7 epidemics were found in Shandong provinces since 2018. One the other hand, vaccination pressure can drive the antigenic evolution of avian influenza viruses such as H5 virus (Peng et al., 2017). More surveillance of the antigenic variation of both H5 and H7 viruses are needed to capture the antigenic variant in time.

A total of 62 H9N2 viruses were sequenced in the study. They were found to be highly similar to each other in all segments. All of these viruses except one isolate belonged to the G57 genotype which has been the dominant genotype of H9N2 viruses circulating in China since 2010. This is consistent with Li’s study which showed that all nine strains of H9N2 viruses isolated in chicken flocks in Shandong province in 2018 belonged to the G57 genotype (Li et al., 2019a). This suggested the great advantage of G57 compared to other genotypes in China. Most newly sequenced H9N2 viruses were similar to those isolated in chickens in mainland China, suggesting the prevalence of H9N2 viruses in chickens.

Molecular markers play important roles in surveillance of emerging influenza viruses, such as monitoring the antigenic variation, drug resistance and host adaptation of the virus (Chen et al., 2006; Liu W. J. et al., 2020). Lots of molecular markers of antigen, host, pathogenicity and drug-resistance have been identified for influenza viruses (Lu et al., 2020; Peng et al., 2020). In the study, all newly sequenced H9N2 viruses harbored several molecular markers associated with human adaptation, mammalian virulence and drug resistance. Although most molecular markers have been experimentally-validated, the role of them in the newly sequenced H9N2 viruses may be changed due to the epistasis (Lyons and Lauring, 2018). Further experiments are needed to validate their role in the newly sequenced H9N2 viruses. Interestingly, the human H9N2 isolate did not have more molecular markers than other viruses, suggesting that the isolate may infect humans accidentally. Nevertheless, more strict protective measures against the H9N2 infection are needed for high-risk people such as poultry worker.

Overall, this study systematically surveyed the AIVs in the LBM of Shandong province from 2013 to 2019 and further revealed the diversity and evolution of H9N2 viruses in the province. It deepens our understanding of the epidemic and evolution of AIVs, and would greatly facilitate the prevention and control of AIVs in China.

## Data availability statement

The datasets presented in this study can be found in online repositories. The names of the repository/repositories and accession number(s) can be found in the article/Supplementary material.

## Ethics statement

The studies involving human participants were reviewed and approved by IRB for Preventive Medicine of Shandong Center for Disease Control and Prevention (reference 2021–24).

## Author contributions

Conceptualization, ZK, ML, TL, and YP: Methodology, TL, YP: Formal Analysis, YP, ML: Investigation, XL, LS and SS: Resources, JL, SL, YH, SZ, ZL, XW and SZ: Writing – Original Draft, TL, YP, ML: Writing – Review and Editing, ZK, TL, YP and ML: Funding Acquisition, TL and YP. All authors listed have made a substantial, direct, and intellectual contribution to the work and approved it for publication.

## Funding

This work was supported by Shandong provincial Natural Science Foundation (ZR2021MH372) and the National Natural Science Foundation of China (32170651).

## Conflict of interest

The authors declare that the research was conducted in the absence of any commercial or financial relationships that could be construed as a potential conflict of interest.

## Publisher’s note

All claims expressed in this article are solely those of the authors and do not necessarily represent those of their affiliated organizations, or those of the publisher, the editors and the reviewers. Any product that may be evaluated in this article, or claim that may be made by its manufacturer, is not guaranteed or endorsed by the publisher.
