# Supplementary material for: Surveillance of avian influenza viruses in live bird markets of Shandong province from 2013 to 2019
Source: Front Microbiol. 2022 Nov 3;13:1030545. doi: 10.3389/fmicb.2022.1030545 (PMC9670132; doi:10.3389/fmicb.2022.1030545)
Supplement: Supplementary file 1 [file Data_Sheet_1.PDF]

Figure S1. The phylogeny of H9N2 viruses on each segment. The newly sequenced H9N2 viruses were colored in red in the trees. The human isolate was marked with blue stars. The clades were labeled on the right side of the tree. The bootstrap value was added on the key nodes, which was generated with 100 bootstrap replicates using MEGA X.

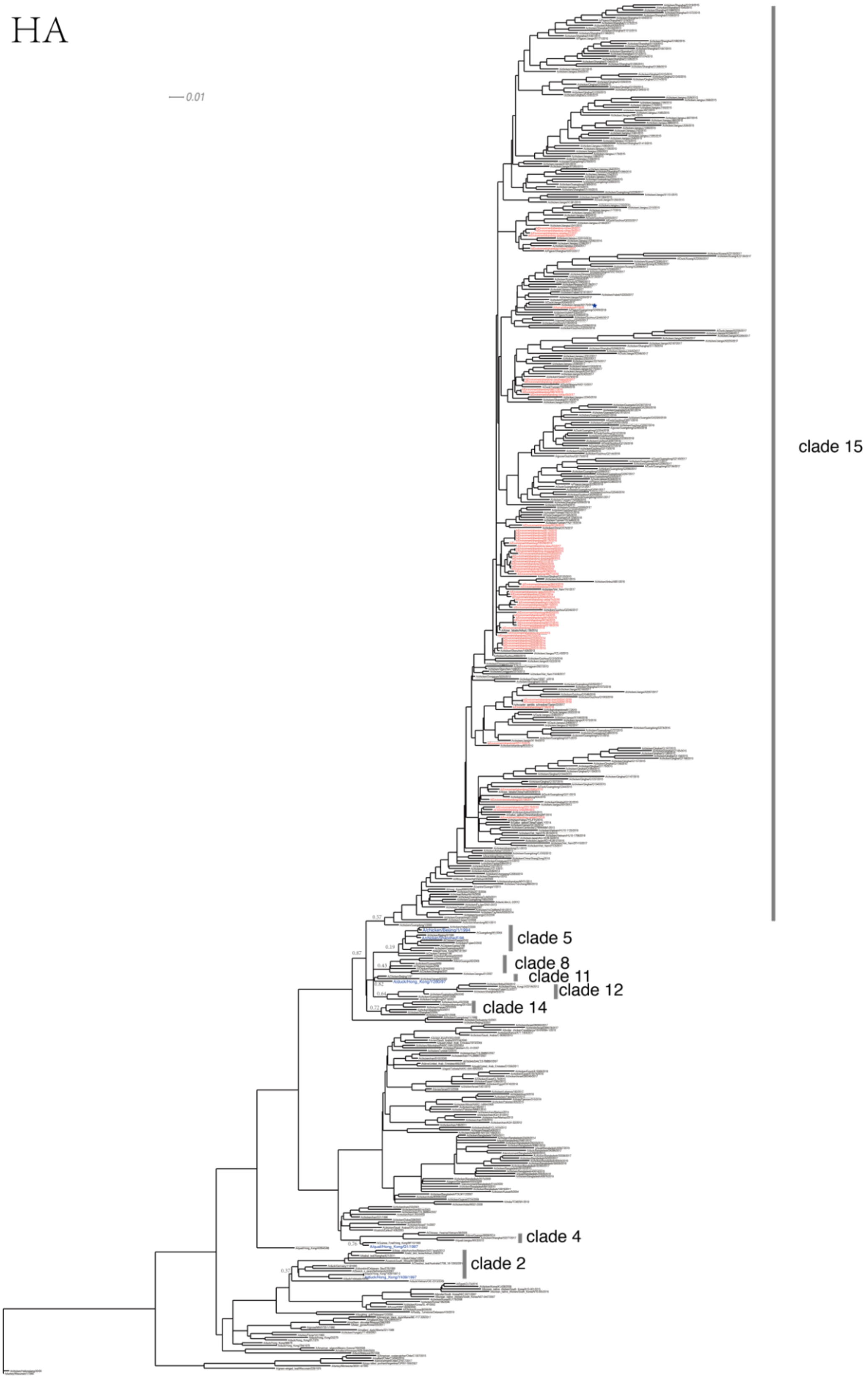

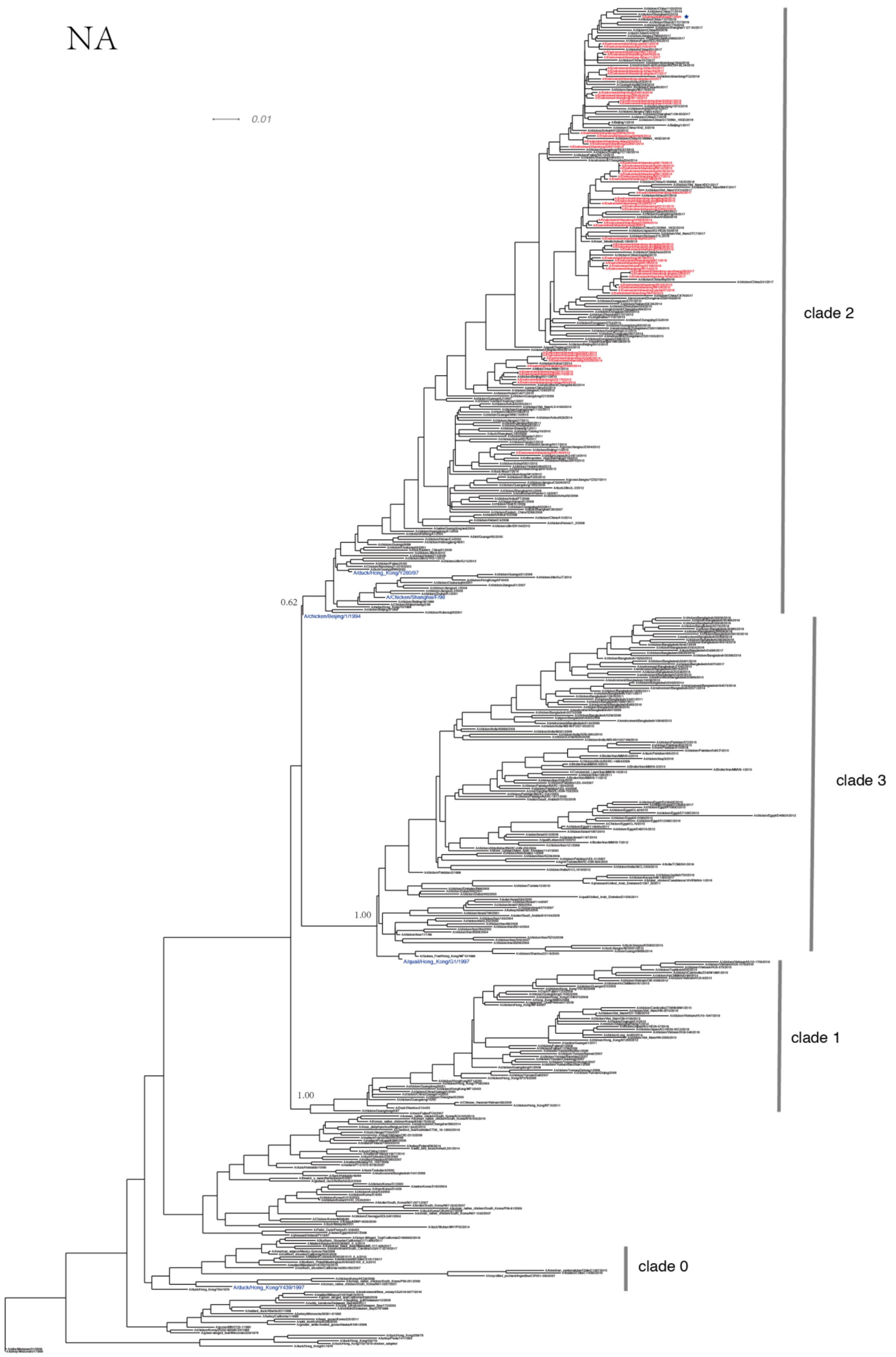
NA
0.01
clade 2
clade 3
clade 1
clade 0
0.62
1.00
1.00
A/duck/Hong_Kong/Y280/97
A/Chicken/Shanghai/F/98
A/chicken/Beijing/1/1994
A/quail/Hong_Kong/G1/1997
A/duck/Hong_Kong/Y439/1997

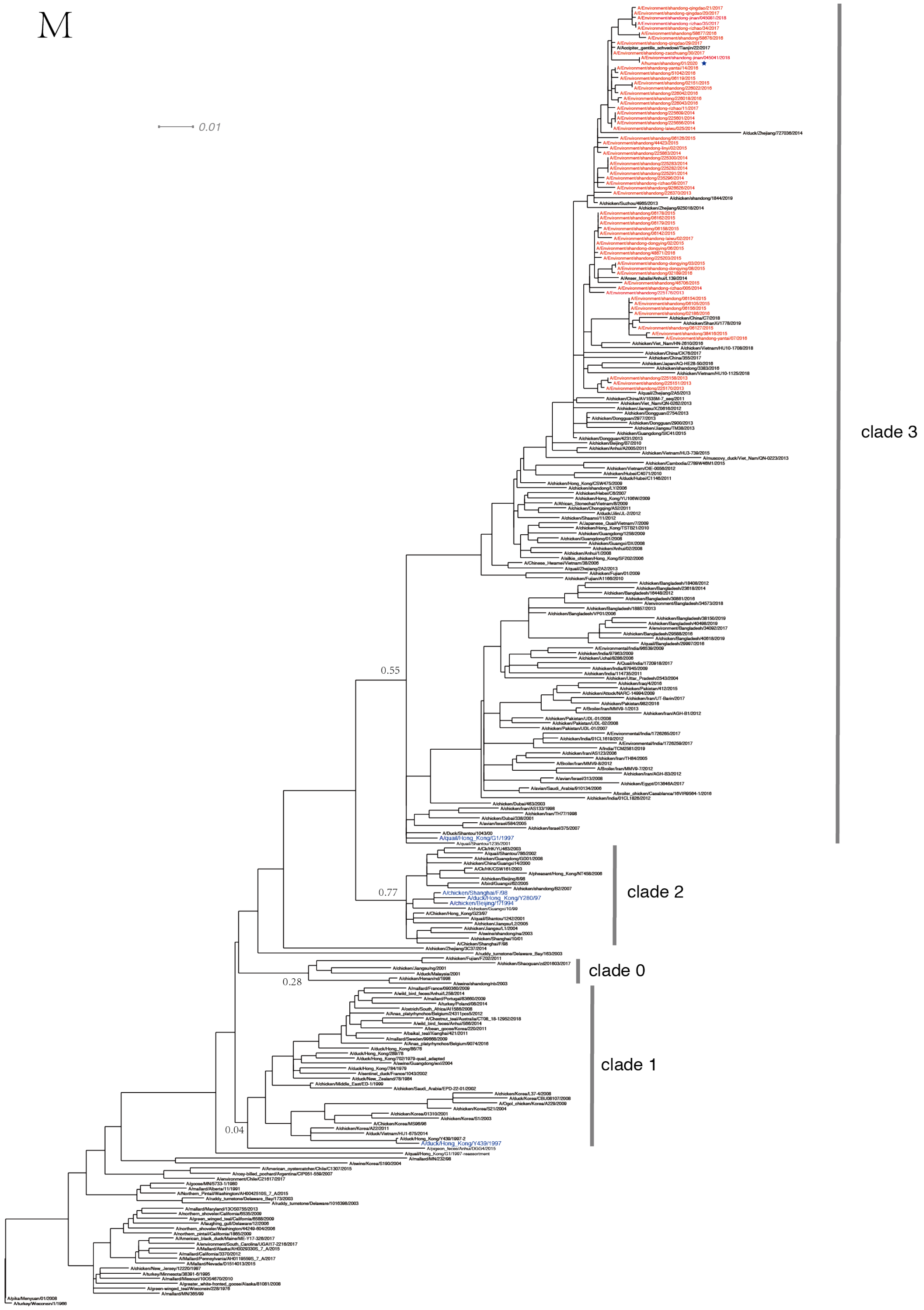
M
0.01
clade 3
clade 2
clade 0
clade 1
0.55
0.77
0.28
0.04

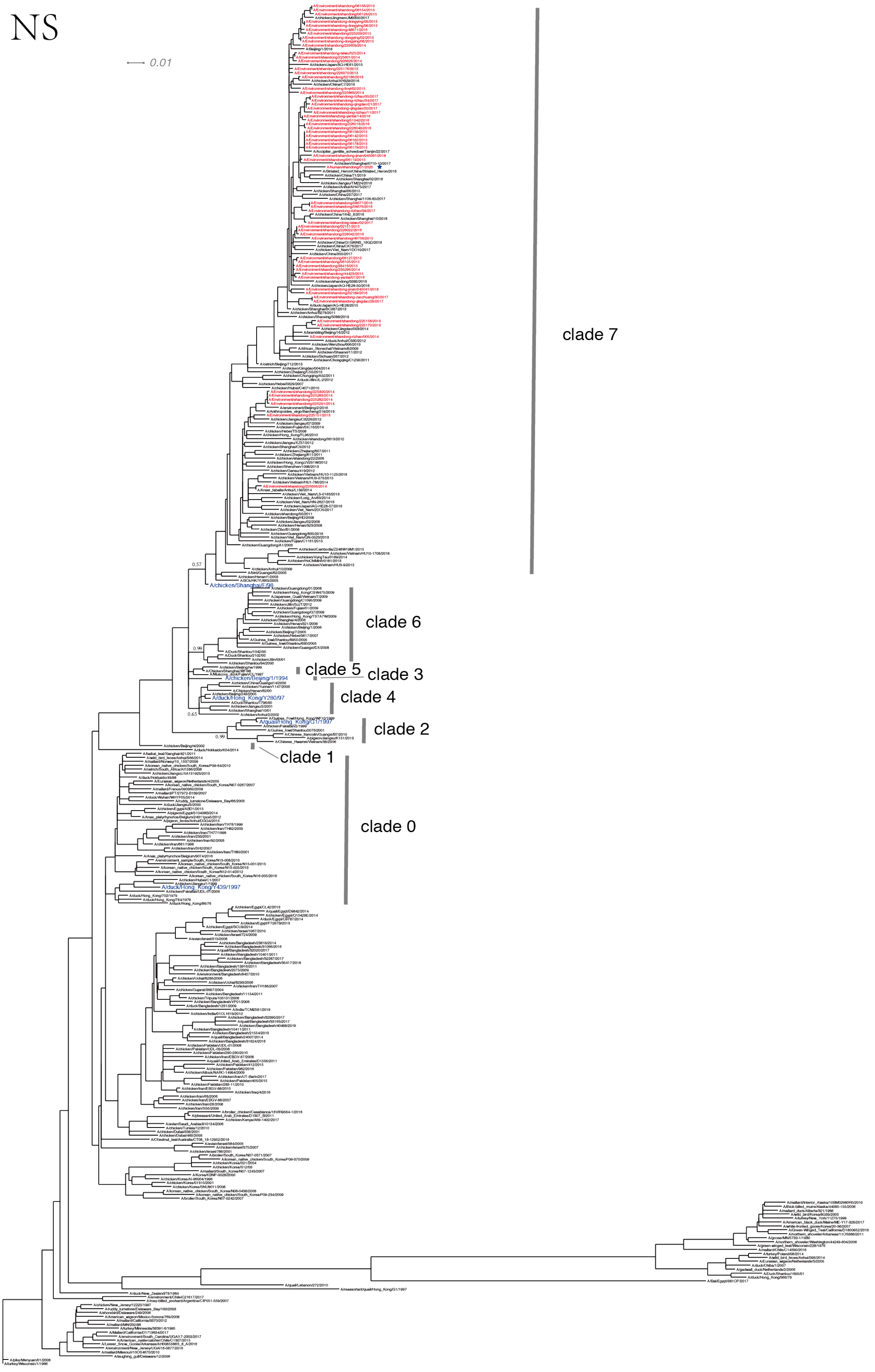
NS
0.01
clade 7
clade 6
clade 5
clade 3
clade 4
clade 2
clade 1
clade 0
0.57
0.99
0.65
0.99

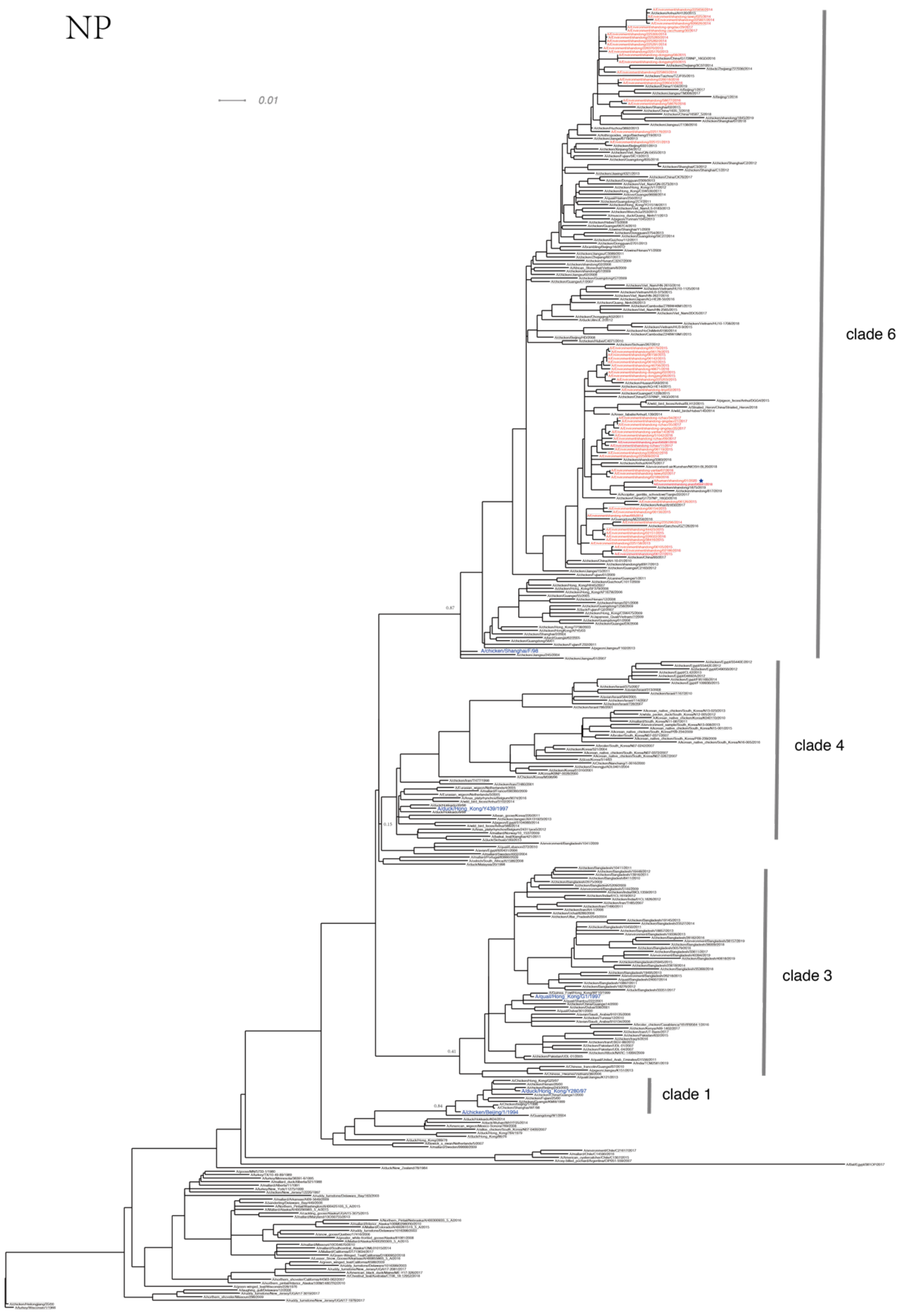
NP
0.01
clade 6
clade 4
clade 3
clade 1
A/chicken/Shanghai/F/98
A/duck/Hong_Kong/Y439/1997
A/quail/Hong_Kong/G1/1997
A/duck/Hong_Kong/Y280/97
A/chicken/Beijing/1/1994

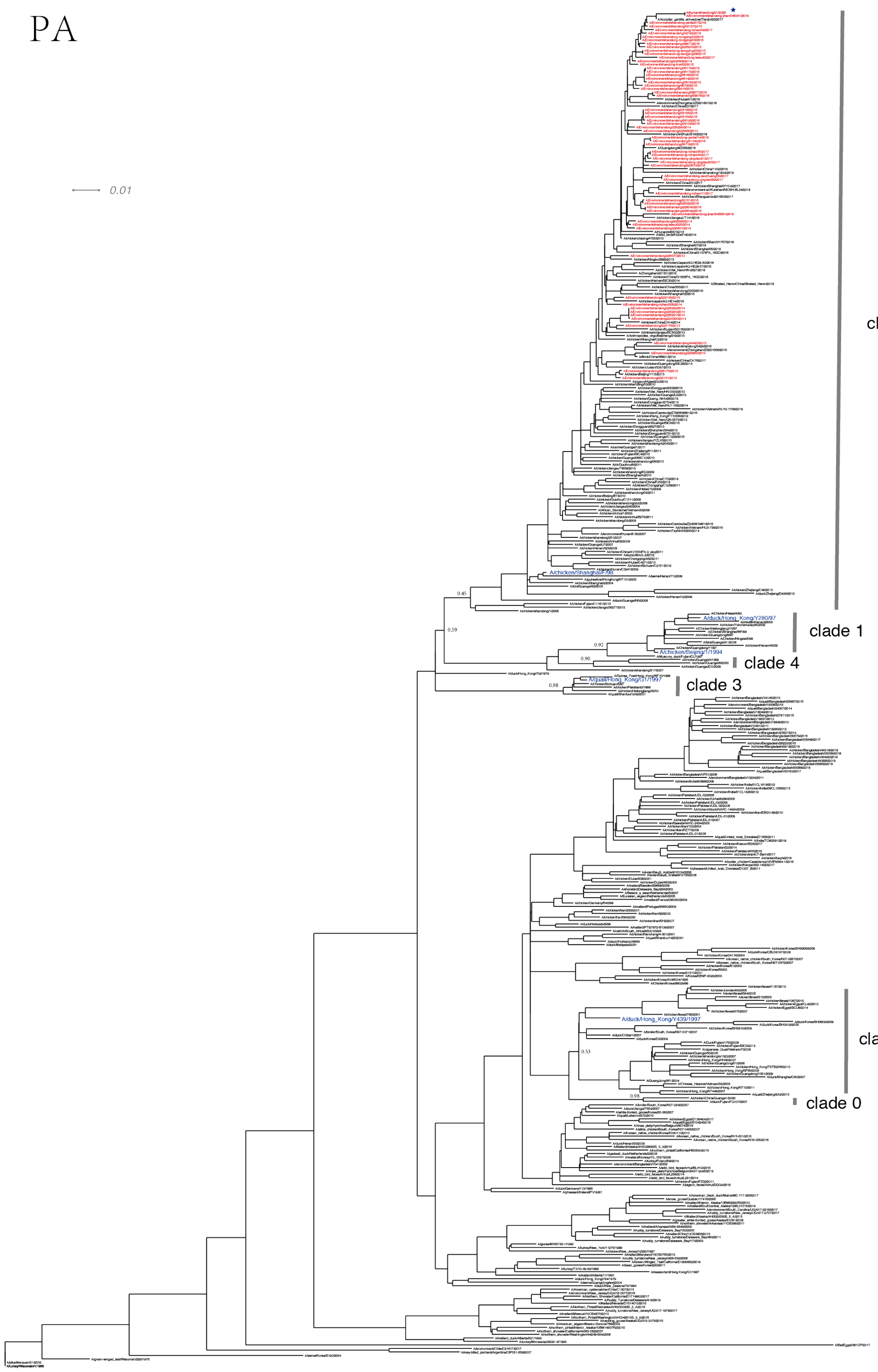
PA
0.01
clade 7
A/chicken/Shanghai/F/98
clade 1
A/duck/Hong_Kong/Y280/97
A/chicken/Beijing/1/1994
clade 4
A/quail/Hong_Kong/G1/1997
clade 3
A/duck/Hong_Kong/Y439/1997
clade 6
clade 0
0.45
0.59
0.92
0.90
0.98
0.53

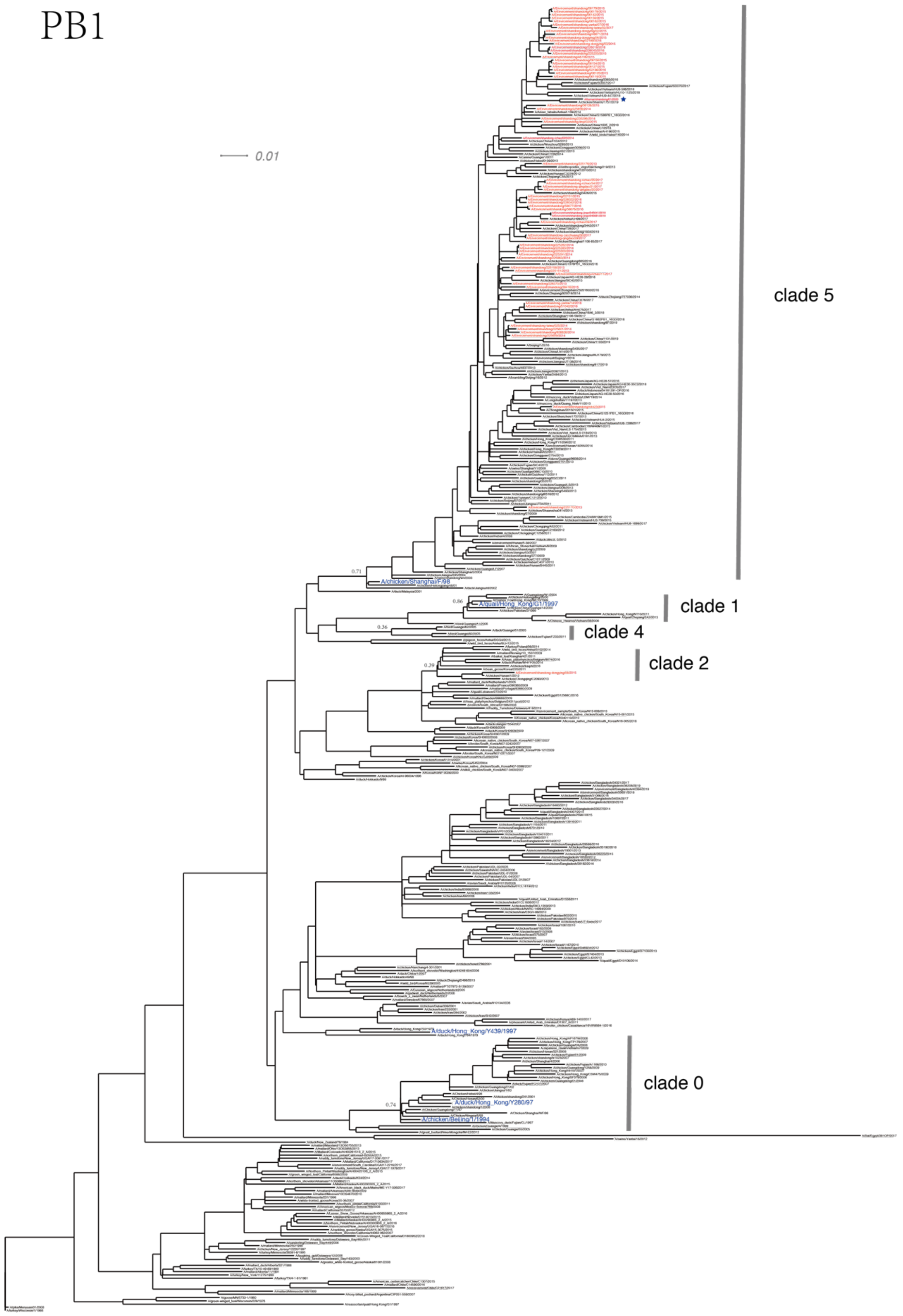
PB1
0.01
clade 5
clade 1
clade 4
clade 2
clade 0
0.71
0.86
0.36
0.39
0.74
A/chicken/Shanghai/F/98
A/quail/Hong_Kong/G1/1997
A/duck/Hong_Kong/Y439/1997
A/duck/Hong_Kong/Y280/97
A/chicken/Beijing/1/1994

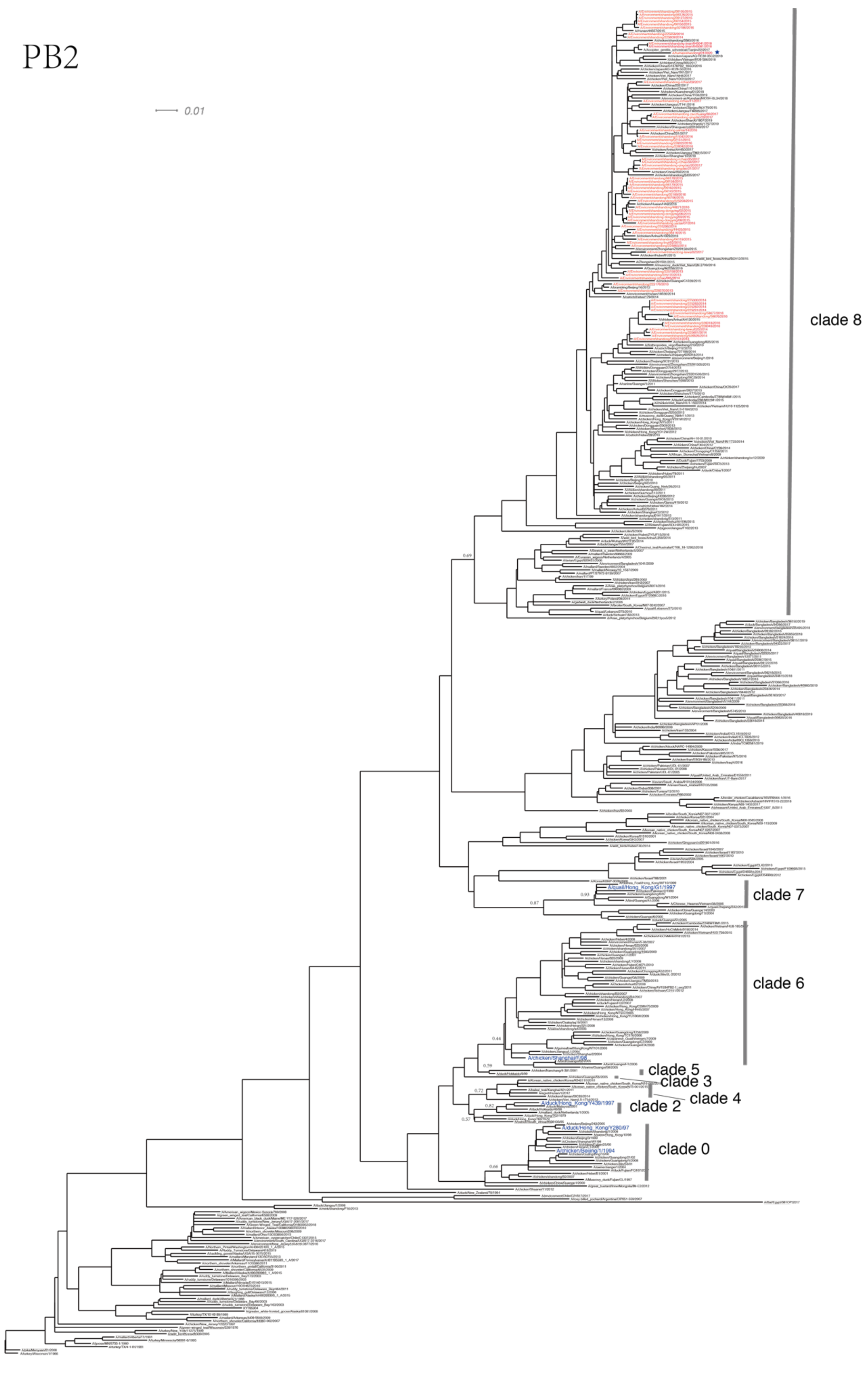

PB2
0.01
clade 8
clade 7
clade 6
clade 5
clade 3
clade 4
clade 2
clade 0
