# Supplementary material for: Surveillance of avian influenza viruses in live bird markets of Shandong province from 2013 to 2019
Source: Front Microbiol. 2022 Nov 3;13:1030545. doi: 10.3389/fmicb.2022.1030545 (PMC9670132; doi:10.3389/fmicb.2022.1030545)
Supplement: Supplementary file 2 [file Data_Sheet_2.docx]

**Supplementary tables**

**Table S1.** The newly sequenced H9N2 viruses

| **Isolate Name** | **Collection date** | **Isolate Id** | **Segment Ids (PB2/PB1/PA/HA/NP/NA/M/NS)** |
| --- | --- | --- | --- |
| A/Environment/shandong-jinan/045041/2018 | 2018-01-25 | 14039058 | EPI2108479/EPI2108480/EPI2108478/EPI2108482/EPI2108475/EPI2108481/EPI2108477/EPI2108476 |
| A/Environment/shandong-jinan/045081/2018 | 2018-02-05 | 14039059 | EPI2108487/EPI2108488/EPI2108486/EPI2108490/EPI2108483/EPI2108489/EPI2108485/EPI2108484 |
| A/human/shandong/01/2020 | 2020-04-20 | 14039060 | EPI2108495/EPI2108496/EPI2108494/EPI2108498/EPI2108491/EPI2108497/EPI2108493/EPI2108492 |
| A/Environment/shandong-linyi/02/2015 | 2015-01-16 | 14039061 | EPI2108503/EPI2108504/EPI2108502/EPI2108506/EPI2108499/EPI2108505/EPI2108501/EPI2108500 |
| A/Environment/shandong/06179/2015 | 2015-12-13 | 14039062 | EPI2108511/EPI2108512/EPI2108510/EPI2108514/EPI2108507/EPI2108513/EPI2108509/EPI2108508 |
| A/Environment/shandong/06178/2015 | 2015-12-13 | 14039063 | EPI2108519/EPI2108520/EPI2108518/EPI2108522/EPI2108515/EPI2108521/EPI2108517/EPI2108516 |
| A/Environment/shandong/06162/2015 | 2015-12-13 | 14039064 | EPI2108527/EPI2108528/EPI2108526/EPI2108530/EPI2108523/EPI2108529/EPI2108525/EPI2108524 |
| A/Environment/shandong/06158/2015 | 2015-12-13 | 14039065 | EPI2108535/EPI2108536/EPI2108534/EPI2108538/EPI2108531/EPI2108537/EPI2108533/EPI2108532 |
| A/Environment/shandong/06156/2015 | 2015-12-13 | 14039066 | EPI2108543/EPI2108544/EPI2108542/EPI2108546/EPI2108539/EPI2108545/EPI2108541/EPI2108540 |
| A/Environment/shandong/06154/2015 | 2015-12-13 | 14039067 | EPI2108551/EPI2108552/EPI2108550/EPI2108554/EPI2108547/EPI2108553/EPI2108549/EPI2108548 |
| A/Environment/shandong/06142/2015 | 2015-11-15 | 14039068 | EPI2108559/EPI2108560/EPI2108558/EPI2108562/EPI2108555/EPI2108561/EPI2108557/EPI2108556 |
| A/Environment/shandong/06127/2015 | 2015-12-13 | 14039069 | EPI2108567/EPI2108568/EPI2108566/EPI2108570/EPI2108563/EPI2108569/EPI2108565/EPI2108564 |
| A/Environment/shandong/06119/2015 | 2015-11-15 | 14039070 | EPI2108575/EPI2108576/EPI2108574/EPI2108578/EPI2108571/EPI2108577/EPI2108573/EPI2108572 |
| A/Environment/shandong/06105/2015 | 2015-12-13 | 14039071 | EPI2108583/EPI2108584/EPI2108582/EPI2108586/EPI2108579/EPI2108585/EPI2108581/EPI2108580 |
| A/Environment/shandong/02189/2016 | 2016-01-08 | 14039072 | EPI2108591/EPI2108592/EPI2108590/EPI2108594/EPI2108587/EPI2108593/EPI2108589/EPI2108588 |
| A/Environment/shandong/02186/2016 | 2016-01-08 | 14039073 | EPI2108599/EPI2108600/EPI2108598/EPI2108602/EPI2108595/EPI2108601/EPI2108597/EPI2108596 |
| A/Environment/shandong/02151/2015 | 2015-10-18 | 14039074 | EPI2108607/EPI2108608/EPI2108606/EPI2108610/EPI2108603/EPI2108609/EPI2108605/EPI2108604 |
| A/Environment/shandong/06126/2015 | 2015-12-13 | 14039075 | EPI2108615/EPI2108616/EPI2108614/EPI2108618/EPI2108611/EPI2108617/EPI2108613/EPI2108612 |
| A/Environment/shandong/46706/2015 | 2015-08-08 | 14039076 | EPI2108623/EPI2108624/EPI2108622/EPI2108626/EPI2108619/EPI2108625/EPI2108621/EPI2108620 |
| A/Environment/shandong/44423/2015 | 2015-08-08 | 14039077 | EPI2108631/EPI2108632/EPI2108630/EPI2108634/EPI2108627/EPI2108633/EPI2108629/EPI2108628 |
| A/Environment/shandong/38416/2015 | 2015-03-16 | 14039078 | EPI2108639/EPI2108640/EPI2108638/EPI2108642/EPI2108635/EPI2108641/EPI2108637/EPI2108636 |
| A/Environment/shandong-dongying/03/2015 | 2015-07-23 | 14039079 | EPI2108647/EPI2108648/EPI2108646/EPI2108650/EPI2108643/EPI2108649/EPI2108645/EPI2108644 |
| A/Environment/shandong-dongying/02/2015 | 2015-06-11 | 14039080 | EPI2108655/EPI2108656/EPI2108654/EPI2108658/EPI2108651/EPI2108657/EPI2108653/EPI2108652 |
| A/Environment/shandong-dongying/06/2015 | 2015-06-11 | 14039081 | EPI2108663/EPI2108664/EPI2108662/EPI2108666/EPI2108659/EPI2108665/EPI2108661/EPI2108660 |
| A/Environment/shandong/225176/2013 | 2013-12-17 | 14039082 | EPI2108671/EPI2108672/EPI2108670/EPI2108674/EPI2108667/EPI2108673/EPI2108669/EPI2108668 |
| A/Environment/shandong/225158/2013 | 2013-12-17 | 14039083 | EPI2108679/EPI2108680/EPI2108678/EPI2108682/EPI2108675/EPI2108681/EPI2108677/EPI2108676 |
| A/Environment/shandong/926626/2014 | 2014-09-23 | 14039084 | EPI2108687/EPI2108688/EPI2108686/EPI2108690/EPI2108683/EPI2108689/EPI2108685/EPI2108684 |
| A/Environment/shandong/225151/2013 | 2013-12-17 | 14039085 | EPI2108695/EPI2108696/EPI2108694/EPI2108698/EPI2108691/EPI2108697/EPI2108693/EPI2108692 |
| A/Environment/shandong/226370/2013 | 2013-12-19 | 14039086 | EPI2108703/EPI2108704/EPI2108702/EPI2108706/EPI2108699/EPI2108705/EPI2108701/EPI2108700 |
| A/Environment/shandong/235296/2014 | 2014-09-24 | 14039087 | EPI2108711/EPI2108712/EPI2108710/EPI2108714/EPI2108707/EPI2108713/EPI2108709/EPI2108708 |
| A/Environment/shandong/226018/2016 | 2016-01-13 | 14039088 | EPI2108719/EPI2108720/EPI2108718/EPI2108722/EPI2108715/EPI2108721/EPI2108717/EPI2108716 |
| A/Environment/shandong/226043/2016 | 2016-01-13 | 14039089 | EPI2108727/EPI2108728/EPI2108726/EPI2108730/EPI2108723/EPI2108729/EPI2108725/EPI2108724 |
| A/Environment/shandong/226042/2016 | 2016-01-13 | 14039090 | EPI2108735/EPI2108736/EPI2108734/EPI2108738/EPI2108731/EPI2108737/EPI2108733/EPI2108732 |
| A/Environment/shandong/226022/2016 | 2016-01-13 | 14039091 | EPI2108743/EPI2108744/EPI2108742/EPI2108746/EPI2108739/EPI2108745/EPI2108741/EPI2108740 |
| A/Environment/shandong-yantai/07/2016 | 2016-04-28 | 14039092 | EPI2108751/EPI2108752/EPI2108750/EPI2108754/EPI2108747/EPI2108753/EPI2108749/EPI2108748 |
| A/Environment/shandong-yantai/14/2016 | 2016-02-17 | 14039093 | EPI2108759/EPI2108760/EPI2108758/EPI2108762/EPI2108755/EPI2108761/EPI2108757/EPI2108756 |
| A/Environment/shandong/225291/2014 | 2014-03-25 | 14039094 | EPI2108767/EPI2108768/EPI2108766/EPI2108770/EPI2108763/EPI2108769/EPI2108765/EPI2108764 |
| A/Environment/shandong/225282/2014 | 2014-03-25 | 14039095 | EPI2108775/EPI2108776/EPI2108774/EPI2108778/EPI2108771/EPI2108777/EPI2108773/EPI2108772 |
| A/Environment/shandong/225300/2014 | 2014-03-25 | 14039096 | EPI2108783/EPI2108784/EPI2108782/EPI2108786/EPI2108779/EPI2108785/EPI2108781/EPI2108780 |
| A/Environment/shandong/225170/2013 | 2013-12-17 | 14039097 | EPI2108791/EPI2108792/EPI2108790/EPI2108794/EPI2108787/EPI2108793/EPI2108789/EPI2108788 |
| A/Environment/shandong-dongying/08/2015 | 2015-03-18 | 14039098 | EPI2108799/EPI2108800/EPI2108798/EPI2108802/EPI2108795/EPI2108801/EPI2108797/EPI2108796 |
| A/Environment/shandong/58677/2016 | 2015-06-08 | 14039099 | EPI2108807/EPI2108808/EPI2108806/EPI2108810/EPI2108803/EPI2108809/EPI2108805/EPI2108804 |
| A/Environment/shandong/58676/2016 | 2016-11-17 | 14039100 | EPI2108815/EPI2108816/EPI2108814/EPI2108818/EPI2108811/EPI2108817/EPI2108813/EPI2108812 |
| A/Environment/shandong/48671/2016 | 2016-03-16 | 14039101 | EPI2108823/EPI2108824/EPI2108822/EPI2108826/EPI2108819/EPI2108825/EPI2108821/EPI2108820 |
| A/Environment/shandong/51042/2016 | 2016-04-08 | 14039102 | EPI2108831/EPI2108832/EPI2108830/EPI2108834/EPI2108827/EPI2108833/EPI2108829/EPI2108828 |
| A/Environment/shandong-laiwu/025/2014 | 2014-03-25 | 14039103 | EPI2108839/EPI2108840/EPI2108838/EPI2108842/EPI2108835/EPI2108841/EPI2108837/EPI2108836 |
| A/Environment/shandong/225203/2015 | 2015-10-15 | 14039104 | EPI2108847/EPI2108848/EPI2108846/EPI2108850/EPI2108843/EPI2108849/EPI2108845/EPI2108844 |
| A/Environment/shandong/225656/2014 | 2014-12-09 | 14039105 | EPI2108855/EPI2108856/EPI2108854/EPI2108858/EPI2108851/EPI2108857/EPI2108853/EPI2108852 |
| A/Environment/shandong/225609/2014 | 2014-11-26 | 14039106 | EPI2108863/EPI2108864/EPI2108862/EPI2108866/EPI2108859/EPI2108865/EPI2108861/EPI2108860 |
| A/Environment/shandong/225863/2014 | 2014-12-19 | 14039107 | EPI2108871/EPI2108872/EPI2108870/EPI2108874/EPI2108867/EPI2108873/EPI2108869/EPI2108868 |
| A/Environment/shandong/225601/2014 | 2014-11-26 | 14039108 | EPI2108879/EPI2108880/EPI2108878/EPI2108882/EPI2108875/EPI2108881/EPI2108877/EPI2108876 |
| A/Environment/shandong/225283/2014 | 2014-05-25 | 14039109 | EPI2108887/EPI2108888/EPI2108886/EPI2108890/EPI2108883/EPI2108889/EPI2108885/EPI2108884 |
| A/Environment/shandong-rizhao/005/2014 | 2014-07-19 | 14039110 | EPI2108895/EPI2108896/EPI2108894/EPI2108898/EPI2108891/EPI2108897/EPI2108893/EPI2108892 |
| A/Environment/shandong-laiwu/02/2017 | 2017-01-08 | 14039111 | EPI2108903/EPI2108904/EPI2108902/EPI2108906/EPI2108899/EPI2108905/EPI2108901/EPI2108900 |
| A/Environment/shandong-rizhao/11/2017 | 2017-01-03 | 14039112 | EPI2108911/EPI2108912/EPI2108910/EPI2108914/EPI2108907/EPI2108913/EPI2108909/EPI2108908 |
| A/Environment/shandong-rizhao/35/2017 | 2017-02-19 | 14039113 | EPI2108919/EPI2108920/EPI2108918/EPI2108922/EPI2108915/EPI2108921/EPI2108917/EPI2108916 |
| A/Environment/shandong-rizhao/34/2017 | 2017-02-19 | 14039114 | EPI2108927/EPI2108928/EPI2108926/EPI2108930/EPI2108923/EPI2108929/EPI2108925/EPI2108924 |
| A/Environment/shandong-zaozhuang/30/2017 | 2017-02-23 | 14039115 | EPI2108935/EPI2108936/EPI2108934/EPI2108938/EPI2108931/EPI2108937/EPI2108933/EPI2108932 |
| A/Environment/shandong-qingdao/29/2017 | 2017-01-18 | 14039116 | EPI2108943/EPI2108944/EPI2108942/EPI2108946/EPI2108939/EPI2108945/EPI2108941/EPI2108940 |
| A/Environment/shandong-qingdao/21/2017 | 2017-01-18 | 14039117 | EPI2108951/EPI2108952/EPI2108950/EPI2108954/EPI2108947/EPI2108953/EPI2108949/EPI2108948 |
| A/Environment/shandong-qingdao/20/2017 | 2017-01-18 | 14039118 | EPI2108959/EPI2108960/EPI2108958/EPI2108962/EPI2108955/EPI2108961/EPI2108957/EPI2108956 |
| A/Environment/shandong-rizhao/09/2017 | 2017-01-03 | 14039119 | EPI2108967/EPI2108968/EPI2108966/EPI2108970/EPI2108963/EPI2108969/EPI2108965/EPI2108964 |

**Table S2**. The surveillance of influenza A viruses in each month. It listed the number of samples and positive samples for influenza A viruses and different subtypes. “-”, not determined.

| Year-  Month | Num of Samples | Num of Positives | H9 | H7 | H5 | H5&H7 | H5&  H9 | H7&  H9 | A  (un-subtyped) |
| --- | --- | --- | --- | --- | --- | --- | --- | --- | --- |
| 201301 | 43 | 0 | - | - | - | - | - | - | - |
| 201302 | 94 | 1 | 1 | - | - | - | - | - | - |
| 201303 | 50 | 0 | - | - | - | - | - | - | - |
| 201304 | 560 | 4 | - | - | - | - | - | 4 | - |
| 201305 | 357 | 1 | 1 | - | - | - | - | - | - |
| 201306 | 31 | 0 | - | - | - | - | - | - | - |
| 201307 | 90 | 0 | - | - | - | - | - | - | - |
| 201308 | 80 | 0 | - | - | - | - | - | - | - |
| 201309 | 41 | 0 | - | - | - | - | - | - | - |
| 201310 | 153 | 30 | 30 | - | - | - | - | - | - |
| 201311 | 336 | 1 | 1 | - | - | - | - | - | - |
| 201312 | 1078 | 34 | 22 | - | 1 | - | 7 | - | 4 |
| 201401 | 830 | 40 | 28 | - | 1 | - | 4 | - | 7 |
| 201402 | 766 | 40 | 20 | - | 15 | - | 4 | 1 | - |
| 201403 | 681 | 18 | 3 | - | - | - | 9 | - | 6 |
| 201404 | 998 | 21 | 1 | 2 | 3 | - | 2 | 2 | 11 |
| 201405 | 662 | 38 | 3 | - | 17 | - | 1 | - | 17 |
| 201406 | 567 | 6 | 1 | - | 2 | - | 1 | - | 2 |
| 201407 | 686 | 16 | 4 | 1 | 2 | - | 2 | 5 | 2 |
| 201408 | 633 | 10 | 9 | - | 1 | - | - | - | - |
| 201409 | 757 | 26 | 14 | - | - | - | - | - | 12 |
| 201410 | 515 | 15 | - | - | 6 | - | - | - | 9 |
| 201411 | 320 | 26 | 6 | - | 6 | - | - | - | 14 |
| 201412 | 547 | 14 | - | - | - | - | - | - | 14 |
| 201501 | 330 | 20 | 15 | - | 2 | - | 3 | - | - |
| 201502 | 153 | 27 | 20 | - | - | - | - | - | 7 |
| 201503 | 302 | 42 | 40 | - | - | - | - | - | 2 |
| 201504 | 319 | 13 | 1 | - | - | - | - | - | 12 |
| 201505 | 151 | 6 | - | - | - | - | - | - | 6 |
| 201506 | 148 | 8 | 2 | - | 2 | - | - | - | 4 |
| 201507 | 171 | 6 | 4 | - | - | - | - | - | 2 |
| 201508 | 99 | 5 | 4 | - | 1 | - | - | - | - |
| 201509 | 401 | 3 | 1 | - | 1 | - | - | - | 1 |
| 201510 | 565 | 22 | 16 | - | 6 | - | - | - | - |
| 201511 | 464 | 15 | 8 | - | 7 | - | - | - | - |
| 201512 | 782 | 80 | 54 | - | 21 | - | 5 | - | - |
| 201601 | 247 | 92 | 55 | 0 | 29 | 0 | 8 | 0 | 0 |
| 201602 | 469 | 107 | 18 | 6 | 23 | 0 | 2 | 57 | 1 |
| 201603 | 544 | 172 | 96 | 0 | 33 | 0 | 11 | 0 | 32 |
| 201604 | 414 | 80 | 49 | 0 | 25 | 0 | 2 | 2 | 2 |
| 201605 | 347 | 38 | 30 | 0 | 7 | 1 | 0 | 0 | 0 |
| 201606 | 274 | 37 | 23 | 0 | 13 | 0 | 1 | 0 | 0 |
| 201607 | 163 | 21 | 8 | 0 | 8 | 0 | 0 | 1 | 4 |
| 201608 | 216 | 8 | 8 | 0 | 0 | 0 | 0 | 0 | 0 |
| 201609 | 259 | 21 | 16 | 0 | 3 | 0 | 2 | 0 | 0 |
| 201610 | 332 | 37 | 35 | 0 | 0 | 0 | 2 | 0 | 0 |
| 201611 | 130 | 14 | 7 | 0 | 5 | 0 | 0 | 0 | 2 |
| 201612 | 383 | 4 | 0 | 0 | 0 | 0 | 0 | 0 | 4 |
| 201701 | 1265 | 342 | 176 | 12 | 9 | 0 | 3 | 72 | 70 |
| 201702 | 909 | 73 | 14 | 15 | 2 | 0 | 0 | 20 | 22 |
| 201703 | 311 | 47 | 35 | 5 | 0 | 0 | 0 | 7 | 0 |
| 201704 | 1866 | 269 | 43 | 165 | 2 | 0 | 0 | 16 | 43 |
| 201705 | 933 | 110 | 49 | 30 | 6 | 0 | 2 | 16 | 7 |
| 201706 | 305 | 9 | 5 | 3 | 0 | 0 | 1 | 0 | 0 |
| 201707 | 120 | 0 | 0 | 0 | 0 | 0 | 0 | 0 | 0 |
| 201708 | 251 | 0 | 0 | 0 | 0 | 0 | 0 | 0 | 0 |
| 201709 | 235 | 22 | 18 | 0 | 0 | 0 | 0 | 0 | 4 |
| 201710 | 265 | 18 | 16 | 0 | 2 | 0 | 0 | 0 | 0 |
| 201711 | 394 | 191 | 186 | 2 | 0 | 0 | 0 | 0 | 3 |
| 201712 | 636 | 33 | 16 | 0 | 5 | 0 | 11 | 0 | 1 |
| 201801 | 753 | 130 | 74 | 5 | 12 | 0 | 16 | 8 | 15 |
| 201802 | 238 | 70 | 56 | 7 | 3 | 1 | 0 | 3 | 0 |
| 201803 | 262 | 45 | 34 | 2 | 3 | 0 | 6 | 0 | 0 |
| 201804 | 135 | 23 | 23 | 0 | 0 | 0 | 0 | 0 | 0 |
| 201805 | 105 | 3 | 1 | 0 | 1 | 0 | 1 | 0 | 0 |
| 201806 | 271 | 12 | 10 | 0 | 0 | 0 | 2 | 0 | 0 |
| 201807 | 196 | 9 | 8 | 0 | 1 | 0 | 0 | 0 | 0 |
| 201808 | 161 | 4 | 4 | 0 | 0 | 0 | 0 | 0 | 0 |
| 201809 | 387 | 16 | 16 | 0 | 0 | 0 | 0 | 0 | 0 |
| 201810 | 295 | 29 | 25 | 4 | 0 | 0 | 0 | 0 | 0 |
| 201811 | 210 | 35 | 30 | 0 | 5 | 0 | 0 | 0 | 0 |
| 201812 | 151 | 35 | 17 | 0 | 8 | 0 | 8 | 0 | 2 |
| 201901 | 236 | 84 | 73 | 0 | 4 | 0 | 7 | 0 | 0 |
| 201902 | 90 | 9 | 9 | 0 | 0 | 0 | 0 | 0 | 0 |
| 201903 | 241 | 50 | 42 | 0 | 2 | 0 | 0 | 0 | 6 |
| 201904 | 136 | 16 | 14 | 0 | 1 | 0 | 1 | 0 | 0 |

**Table S3**. The genotype of each newly sequenced H9N2 virus isolate.

| H9N2 virus isolate | HA | NA | M | NS | NP | PA | PB1 | PB2 | Genotype |
| --- | --- | --- | --- | --- | --- | --- | --- | --- | --- |
| A/Environment/shandong-jinan/045041/2018 | 15 | 2 | 3 | 7 | 6 | 7 | 5 | 8 | G57 |
| A/Environment/shandong-jinan/045081/2018 | 15 | 2 | 3 | 7 | 6 | 7 | 5 | 8 | G57 |
| A/human/shandong/01/2020 | 15 | 2 | 3 | 7 | 6 | 7 | 5 | 8 | G57 |
| A/Environment/shandong-linyi/02/2015 | 15 | 2 | 3 | 7 | 6 | 7 | 5 | 8 | G57 |
| A/Environment/shandong/06179/2015 | 15 | 2 | 3 | 7 | 6 | 7 | 5 | 8 | G57 |
| A/Environment/shandong/06178/2015 | 15 | 2 | 3 | 7 | 6 | 7 | 5 | 8 | G57 |
| A/Environment/shandong/06162/2015 | 15 | 2 | 3 | 7 | 6 | 7 | 5 | 8 | G57 |
| A/Environment/shandong/06158/2015 | 15 | 2 | 3 | 7 | 6 | 7 | 5 | 8 | G57 |
| A/Environment/shandong/06156/2015 | 15 | 2 | 3 | 7 | 6 | 7 | 5 | 8 | G57 |
| A/Environment/shandong/06154/2015 | 15 | 2 | 3 | 7 | 6 | 7 | 5 | 8 | G57 |
| A/Environment/shandong/06142/2015 | 15 | 2 | 3 | 7 | 6 | 7 | 5 | 8 | G57 |
| A/Environment/shandong/06127/2015 | 15 | 2 | 3 | 7 | 6 | 7 | 5 | 8 | G57 |
| A/Environment/shandong/06119/2015 | 15 | 2 | 3 | 7 | 6 | 7 | 5 | 8 | G57 |
| A/Environment/shandong/06105/2015 | 15 | 2 | 3 | 7 | 6 | 7 | 5 | 8 | G57 |
| A/Environment/shandong/02189/2016 | 15 | 2 | 3 | 7 | 6 | 7 | 5 | 8 | G57 |
| A/Environment/shandong/02186/2016 | 15 | 2 | 3 | 7 | 6 | 7 | 5 | 8 | G57 |
| A/Environment/shandong/02151/2015 | 15 | 2 | 3 | 7 | 6 | 7 | 5 | 8 | G57 |
| A/Environment/shandong/06126/2015 | 15 | 2 | 3 | 7 | 6 | 7 | 5 | 8 | G57 |
| A/Environment/shandong/46706/2015 | 15 | 2 | 3 | 7 | 6 | 7 | 5 | 8 | G57 |
| A/Environment/shandong/44423/2015 | 15 | 2 | 3 | 7 | 6 | 7 | 5 | 8 | G57 |
| A/Environment/shandong/38416/2015 | 15 | 2 | 3 | 7 | 6 | 7 | 5 | 8 | G57 |
| A/Environment/shandong-dongying/03/2015 | 15 | 2 | 3 | 7 | 6 | 7 | 5 | 8 | G57 |
| A/Environment/shandong-dongying/02/2015 | 15 | 2 | 3 | 7 | 6 | 7 | 5 | 8 | G57 |
| A/Environment/shandong-dongying/06/2015 | 15 | 2 | 3 | 7 | 6 | 7 | 5 | 8 | G57 |
| A/Environment/shandong/225176/2013 | 15 | 2 | 3 | 7 | 6 | 7 | 5 | 8 | G57 |
| A/Environment/shandong/225158/2013 | 15 | 2 | 3 | 7 | 6 | 7 | 5 | 8 | G57 |
| A/Environment/shandong/926626/2014 | 15 | 2 | 3 | 7 | 6 | 7 | 5 | 8 | G57 |
| A/Environment/shandong/225151/2013 | 15 | 2 | 3 | 7 | 6 | 7 | 5 | 8 | G57 |
| A/Environment/shandong/226370/2013 | 15 | 2 | 3 | 7 | 6 | 7 | 5 | 8 | G57 |
| A/Environment/shandong/235296/2014 | 15 | 2 | 3 | 7 | 6 | 7 | 5 | 8 | G57 |
| A/Environment/shandong/226018/2016 | 15 | 2 | 3 | 7 | 6 | 7 | 5 | 8 | G57 |
| A/Environment/shandong/226043/2016 | 15 | 2 | 3 | 7 | 6 | 7 | 5 | 8 | G57 |
| A/Environment/shandong/226042/2016 | 15 | 2 | 3 | 7 | 6 | 7 | 5 | 8 | G57 |
| A/Environment/shandong/226022/2016 | 15 | 2 | 3 | 7 | 6 | 7 | 5 | 8 | G57 |
| A/Environment/shandong-yantai/07/2016 | 15 | 2 | 3 | 7 | 6 | 7 | 5 | 8 | G57 |
| A/Environment/shandong-yantai/14/2016 | 15 | 2 | 3 | 7 | 6 | 7 | 5 | 8 | G57 |
| A/Environment/shandong/225291/2014 | 15 | 2 | 3 | 7 | 6 | 7 | 5 | 8 | G57 |
| A/Environment/shandong/225282/2014 | 15 | 2 | 3 | 7 | 6 | 7 | 5 | 8 | G57 |
| A/Environment/shandong/225300/2014 | 15 | 2 | 3 | 7 | 6 | 7 | 5 | 8 | G57 |
| A/Environment/shandong/225170/2013 | 15 | 2 | 3 | 7 | 6 | 7 | 5 | 8 | G57 |
| A/Environment/shandong-dongying/08/2015 | 15 | 2 | 3 | 7 | 6 | 7 | 2 | 8 | G69 |
| A/Environment/shandong/58677/2016 | 15 | 2 | 3 | 7 | 6 | 7 | 5 | 8 | G57 |
| A/Environment/shandong/58676/2016 | 15 | 2 | 3 | 7 | 6 | 7 | 5 | 8 | G57 |
| A/Environment/shandong/48671/2016 | 15 | 2 | 3 | 7 | 6 | 7 | 5 | 8 | G57 |
| A/Environment/shandong/51042/2016 | 15 | 2 | 3 | 7 | 6 | 7 | 5 | 8 | G57 |
| A/Environment/shandong-laiwu/025/2014 | 15 | 2 | 3 | 7 | 6 | 7 | 5 | 8 | G57 |
| A/Environment/shandong/225203/2015 | 15 | 2 | 3 | 7 | 6 | 7 | 5 | 8 | G57 |
| A/Environment/shandong/225656/2014 | 15 | 2 | 3 | 7 | 6 | 7 | 5 | 8 | G57 |
| A/Environment/shandong/225609/2014 | 15 | 2 | 3 | 7 | 6 | 7 | 5 | 8 | G57 |
| A/Environment/shandong/225863/2014 | 15 | 2 | 3 | 7 | 6 | 7 | 5 | 8 | G57 |
| A/Environment/shandong/225601/2014 | 15 | 2 | 3 | 7 | 6 | 7 | 5 | 8 | G57 |
| A/Environment/shandong/225283/2014 | 15 | 2 | 3 | 7 | 6 | 7 | 5 | 8 | G57 |
| A/Environment/shandong-rizhao/005/2014 | 15 | 2 | 3 | 7 | 6 | 7 | 5 | 8 | G57 |
| A/Environment/shandong-laiwu/02/2017 | 15 | 2 | 3 | 7 | 6 | 7 | 5 | 8 | G57 |
| A/Environment/shandong-rizhao/11/2017 | 15 | 2 | 3 | 7 | 6 | 7 | 5 | 8 | G57 |
| A/Environment/shandong-rizhao/35/2017 | 15 | 2 | 3 | 7 | 6 | 7 | 5 | 8 | G57 |
| A/Environment/shandong-rizhao/34/2017 | 15 | 2 | 3 | 7 | 6 | 7 | 5 | 8 | G57 |
| A/Environment/shandong-zaozhuang/30/2017 | 15 | 2 | 3 | 7 | 6 | 7 | 5 | 8 | G57 |
| A/Environment/shandong-qingdao/29/2017 | 15 | 2 | 3 | 7 | 6 | 7 | 5 | 8 | G57 |
| A/Environment/shandong-qingdao/21/2017 | 15 | 2 | 3 | 7 | 6 | 7 | 5 | 8 | G57 |
| A/Environment/shandong-qingdao/20/2017 | 15 | 2 | 3 | 7 | 6 | 7 | 5 | 8 | G57 |
| A/Environment/shandong-rizhao/09/2017 | 15 | 2 | 3 | 7 | 6 | 7 | 5 | 8 | G57 |

**Table S4**. The drug-resistance associated molecular markers identified in the newly sequenced H9N2 virus strains. Those which were found in more than 50 viruses were highlighted in bold.

| **Protein** | **Mutation** | **Ratio** | **Mutation effect** |
| --- | --- | --- | --- |
| NA | **151D** | 62/62 | oseltamivir/zanamivir drug resistance |
|  | 292K | 1/62 | oseltamivir/zanamivir drug resistance |
| M2 | **21G** | 60/62 | amantadine drug resistance |
|  | **31N** | 62/62 | amantadine drug resistance |
